# Impact of the sinus node recovery time after termination of atrial fibrillation during catheter ablation on clinical outcomes in patients with persistent atrial fibrillation

**DOI:** 10.1371/journal.pone.0259750

**Published:** 2021-11-05

**Authors:** Keita Watanabe, Yasutoshi Nagata, Giichi Nitta, Shinichiro Okata, Masashi Nagase, Ryoichi Miyazaki, Sho Nagamine, Masakazu Kaneko, Tetsumin Lee, Toshihiro Nozato, Takashi Ashikaga, Masahiko Goya, Tetsuo Sasano

**Affiliations:** 1 Department of Cardiology, Japanese Red Cross Musashino Hospital, Kyonancho, Musashino city, Tokyo, Japan; 2 Department of Cardiology, Tokyo Medical and Dental University, Yushima, Bunkyo-ku, Tokyo, Japan; Ohio State University, UNITED STATES

## Abstract

**Background:**

Although long sinus arrest is occasionally observed during atrial fibrillation (AF) catheter ablation when the fibrillation was terminated, its meaning and prognosis have not yet been clearly elucidated. We hypothesized that sinus node recovery time (SNRT) after termination of AF (time from termination of AF to the earliest sinus node activation) could reflect the extent of atrial remodeling, influencing the formation of non-pulmonary vein (non-PV) triggers and post-ablation outcomes.

**Method:**

The participants were 157 consecutive patients with persistent AF (male: 77.1%, age: 63.3±11.2 years) who underwent catheter ablation. We recorded SNRT after terminating AF by radiofrequency delivery or electrical cardioversion during the first ablation and evaluated the relationships between SNRT and atrial tachyarrhythmia recurrence and between SNRT and non-PV triggers after repeat ablation.

**Results:**

Forty-five patients (28.7%) experienced recurrence of atrial tachyarrhythmias. Patients with recurrence had longer SNRTs (1738 ms vs. 1394 ms, p = 0.012). In the multivariate logistic regression analysis, only SNRT ≥2128ms was a significant independent predictor of clinical AF recurrence (hazard ratio 7.48; 95% confidence interval 2.94–19.00; P<0.001). Kaplan–Meier estimator showed that the recurrence-free rate was significantly lower if ≥ 2128ms (log-rank, p<0.001). Thirty-five patients (77.8%) underwent a second ablation. Although there was no difference in the rate of pulmonary vein reconnections (78.6% vs. 71.4%, p = 0.712), non-PV triggers were observed more frequently in the longer SNRT group (57.1% vs. 14.3%, p = 0.012).

**Conclusions:**

Patients with a prolonged SNRT had a higher prevalence of AF recurrence after the first ablation and higher inducibility of non-PV triggers. Measuring SNRT might be used for the stratification of patients with persistent AF.

## Introduction

Long sinus arrest is occasionally observed during atrial fibrillation (AF) catheter ablation when the fibrillation is terminated. Although the precise mechanism of this phenomenon is uncertain, it has been suggested that sinus node dysfunction, caused by atrial electric remodeling, may be a contributing factor [1]. Recently, AF catheter ablation has been performed on elderly patients [2], and the relationship between sinus node dysfunction and AF is more in demand. The relationship has been described using late gadolinium-enhanced magnetic resonance imaging (LGE-MRI) [3] and atrial voltage mapping during ablation [4], and has been associated with atrial remodeling through myocardial fibrosis and reduced electrical voltage. In these reports, the degree of damage in the right and left atria is related. Some papers have reported that atrial remodeling may proceeds diffusely in both atria, rather than locally [3,5].

We suspect that if the remodeling occurs diffusely, it will affect not only the left but also the right atria, as well as the sinus node. We hypothesized that the sinus node recovery time (SNRT) after termination of AF (time from AF termination to the earliest sinus node activation) reflects the extent of atrial remodeling, which in turn influences non-pulmonary vein (non-PV) trigger formations and hence ablation outcome.

## Materials and methods

This was a retrospective single-center observational study consisting of two parts. First, we examined the relationship between the SNRT recorded during the initial ablation and clinical outcomes; second, we examined the relationship between SNRT and the presence of non-PV triggers in recurrent cases.

### Study population

We identified and included all the patients with persistent and longstanding persistent AF who underwent an initial ablation between January 2015 and December 2018 at the Japanese Red Cross Musashino Hospital. Longstanding persistent AF was defined as AF lasting for more than one year. Patients who were observed sinus rhythm before catheter ablation were excluded as paroxysmal atrial fibrillation.

This study was approved by the institutional review board of the Japanese Red Cross Musashino Hospital, and this study complied with the ethical principles of the Declaration of Helsinki and the Japanese Ethical Guideline for Medical and Health Research Involving Human Subjects. All participants were notified that they would be included in the study, and we explained to them that they were free to opt out of participation at any time.

### Strategy for the initial ablation

Echocardiography and three-dimensional (3D) contrast-enhanced computed tomography were performed within a month before admission. All antiarrhythmic drugs except β-blockers were discontinued a week before ablation. Vascular access was achieved with sheaths placed via the jugular and femoral veins. An internal cardioversion system was inserted from the jugular vein. The system consisted of a BeeAT catheter (Japan Lifeline, Tokyo, Japan) and a dedicated defibrillator (Shock AT, Japan Lifeline), which together constitute the only internal cardioversion system currently approved for use in Japan. The BeeAT catheter has 20 poles, which can record local electrograms from the coronary sinus, cavo-tricuspid isthmus, right atria, and superior vena cava (SVC). Long sheaths (Agilis and SL0, Abbott, St. Paul, MN, USA) were inserted via the femoral vein. An activated clotting time of 300–350 seconds was maintained with a continuous infusion of heparin during the procedure. After transseptal puncture, the two long sheaths were introduced into both superior PVs via the same transseptal insertion site, and pulmonary venography was performed. A 3D mapping system and an irrigated tip ablation catheter were used in all cases. Multipolar circular catheters (EPstar Libero, Japan Lifeline, Tokyo, Japan) were placed at the PV ostium. Catheter ablation was started during AF, and extensive encircling pulmonary vein isolation (EEPVI) was performed in all cases. Radiofrequency energy was delivered at 25–30 W and a cut-off temperature of 45°C. If non-PV triggers from the SVC were observed during isoproterenol provocation, SVC isolation was performed after EEPVI. Additional procedures, such as SVC isolation, were left to the discretion of each physician. No substrate modification, such as complex fractionated atrial electrogram (CFAE) ablation, was performed.

### Follow-up

Close clinical follow-up (2 weeks, 1 month, and then every 2 to 4 months) with 12-lead electrocardiograms at each visit was arranged. Twenty-four-hour Holter monitoring or event recording was performed at least at 3 and 12 months. If patients experienced palpitations, 24-hour Holter monitoring or event recording was performed repeatedly to define the cause of the tachycardia, including at 6 and 9 months. Recurrence was defined as any sustained atrial tachyarrhythmia longer than 30 seconds after a blanking period of 3 months after the procedure.

### Second ablation

If AF recurrence was observed during follow-up, we actively encouraged the patient to undergo a second ablation. The catheters were inserted as in the first procedure. If an electrical reconnection of the PV was observed using a multipolar circular catheter, PV re-isolation was performed. After the completion of PV isolation, we attempted to induce AF with an intravenous isoproterenol infusion (up to 5 μg/min) and atrial burst pacing. After repeated AF induction and defibrillation, the subsequent emergence of a non-PV trigger was mapped to its origin with a circular catheter, and focal ablation was performed for the non-PV trigger areas. When non-PV triggers were located in the SVC, electrical isolation was performed. In the case of unmappable non-PV triggers, substrate modification such as CFAE ablation and left atrial posterior wall isolation was added at the discretion of the operator. PV triggers were defined as frequent dissociation spikes or fibrillation in the re-connected PVs. Non-PV triggers were determined by the origin of AF from sites other than the PVs.

### Measurement of the sinus node recovery time

**Fig 1:**
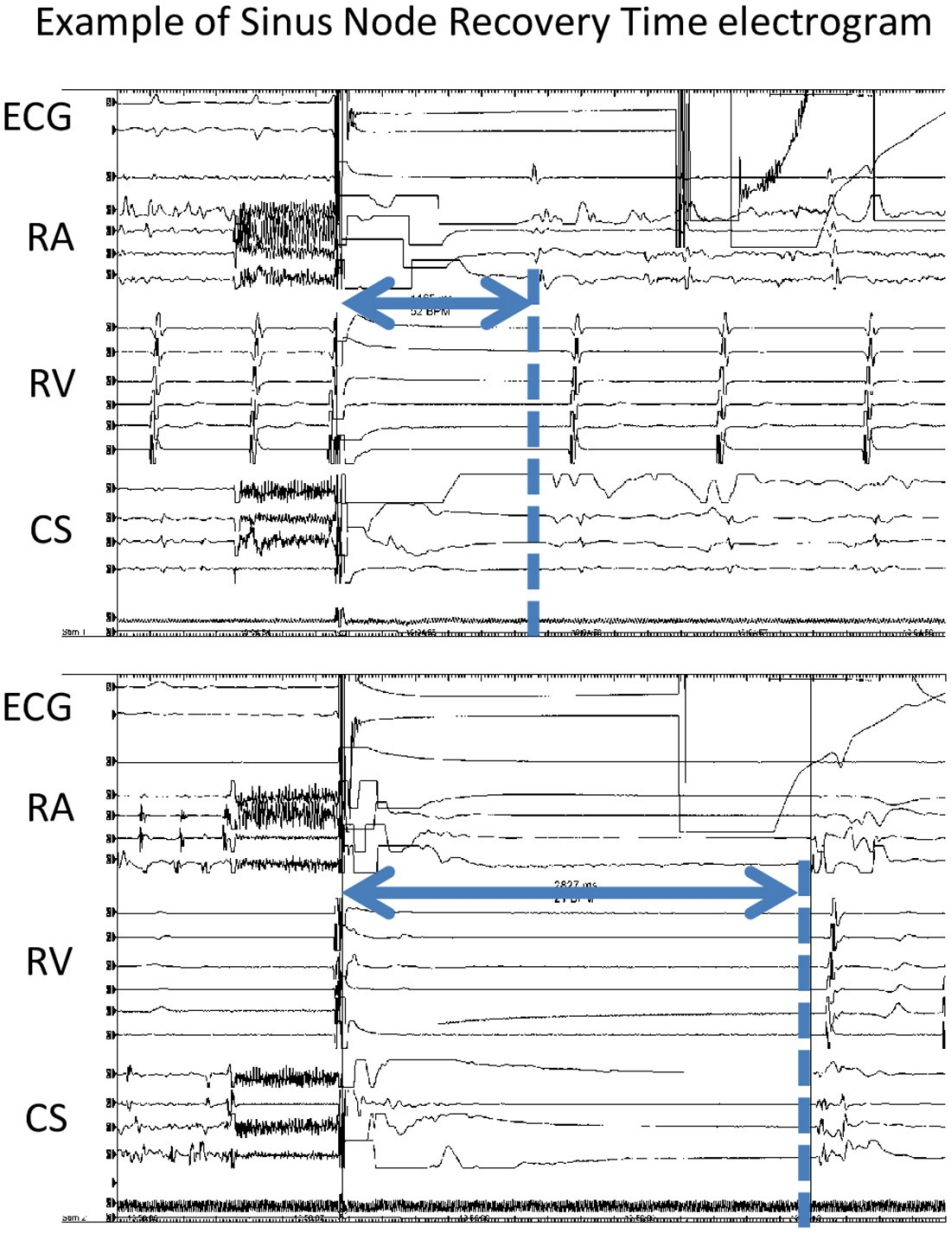
Measurement of the sinus node recovery time. RA, right atria; RV, right ventricular; CS, coronary sinus.

We measured the SNRT recorded during radiofrequency delivery or electrical cardioversion during the first ablation session. If the AF was terminated by radiofrequency delivery, the SNRT was defined as the time interval between the last atrial activation recorded on the intracardiac electrocardiogram and the sinus node activation time. If AF persisted after the completion of PV isolation, internal cardioversion was performed. We initiated the shock energy from 5 J to 30 J (serial shocks 5 J, 10 J, 20 J, and 30 J). If the AF was not terminated by internal cardioversion, external cardioversion was considered. We defined the SNRT as the time interval between the internal electrical cardioversion and the earliest sinus node activation (Fig 1). Sinus rhythm was determined by the excitation sequence of the intracardiac electrocardiogram. Patients whose SNRTs were recorded multiple times had their first SNRT used.

### Statistical analysis

Differences in baseline clinical characteristics and echocardiographic parameters were compared using Student’s t-test for parametric data and Mann-Whitney U tests for non-parametric data. Categorical variables were compared using the chi-square test or Fisher’s exact test. Receiver operating characteristic (ROC) curves were generated to determine the optimal predictive cut-off values with sensitivity and specificity to discriminate patients according to the risk of recurrence. Univariate logistic regression analysis was used to assess the prognostic value of baseline clinical characteristics and clinical termination features. Variables with a p-value <0.05 in the univariate analysis, together with previously reported factors, were selected for further multivariate analysis to examine the predictive effect of each factor on the risk of recurrence.

All statistical analyses were performed using EZR software (Saitama Medical Center, Jichi Medical University, Saitama, Japan) [6], which is a graphical user interface for R (The R Foundation for Statistical Computing, Vienna, Austria). More precisely, it is a modified version of R commander designed to add statistical functions frequently used in biostatistics.

## Results

### Clinical characteristics and clinical recurrence

A total of 167 consecutive patients with non-paroxysmal AF were initially included in this study. We excluded 10 patients, either at the patient’s request or because the SNRT could not be measured (because of interference on the intracardiac electrocardiogram or pacing).

Therefore, 157 patients (age, 63.3±11.2 years; 121 males; left atrial diameter, 41.1± 5.7mm) were finally included. The baseline clinical characteristics are presented in Table 1. After a mean follow-up period of 523 days (range, 382–742 days), 45 patients (28.7%) experienced recurrence of atrial tachyarrhythmias. Pre-procedural clinical characteristics had no effect on recurrence. There was no difference in AF recurrence regardless of whether AF was terminated by radiofrequency deliveries or electrical cardioversion (13.5% vs. 23.8%, p = 0.242). In the Holter ECG at 3 months after catheter ablation, the recurrence group had significantly higher premature atrial contractions (232 vs. 120, p = 0.004) and tended to have a higher total heart rate (97053 vs. 102712, p = 0.079).

**Table 1:** Patient characteristics and comparison between clinical recurrence and no clinical recurrence.

	Total		Clinical recurrence		No clinical recurrence		p-value
	N = 157		N = 45		N = 112		
Baseline findings							
Age	63.3	±11.2	63.7	±12.2	63.2	±10.8	0.781
Male	121/157	77.1%	31/45	68.9%	90/112	80.4%	0.143
BMI	24.6	±3.5	24.5	±4.1	24.7	±3.2	0.738
CHADS2	1.1	±1.1	1.2	±1.1	1.1	±1.0	0.690
CHA2DS2-VASc	1.8	±1.5	2	±1.6	1.8	±1.4	0.415
HF	37/157	23.6%	9/45	20.0%	26/112	23.2%	0.832
HTN	68/157	43.3%	22/45	48.9%	46/112	41.1%	0.380
DM	24/157	15.3%	5/45	11.1%	19/112	17.0%	0.465
Stroke	9/157	5.7%	2/45	4.4%	7/112	6.3%	1
CRF	10/157	6.4%	3/45	6.7%	7/112	6.3%	1
SSS	13/157	8.3%	5/45	11.1%	8/112	7.1%	0.520
SAS	6/157	3.8%	1/45	2.2%	5/112	4.5%	0.674
long persistent	40/157	25.5%	15/45	33.3%	25/112	22.3%	0.162
Medication before ablation							
ACE-I ARB	48/157	30.6%	9/45	20.0%	39/112	34.8%	0.085
βblocker	77/157	49.0%	21/45	46.7%	56/112	50.0%	0.727
AAD	31/157	19.7%	13/45	28.9%	18/112	16.1%	0.079
Echocardiographic findings							
LAD	41.1	±5.7	41.0	±5.6	41.2	±5.8	0.885
LVEF	62.4	±11.8	62.5	±9.4	62.3	±12.7	0.908
E/e’	5.8	±2.1	6.0	±2.8	5.7	±1.7	0.405
Clinical findings during ablation							
SNRT	1447	(1228–1899)	1738	(1293–2516)	1394	(1224–1719)	0.012
Radiofrequency termination	29/138	21.0%	5/37	13.5%	24/101	23.8%	0.242
shock energy	13	±7.3	14.2	±8.2	12.5	±6.9	0.288
Number of shocks	1.9	±1.4	2.1	±1.3	1.8	±1.4	0.396
SVC isolation	34/157	21.7%	8/45	17.8%	26/112	23.2%	0.525
Non-PV ablation without SVC	6/157	3.8%	3/45	6.7%	3/109	2.8%	0.355
Holter ECG at 3 months							
total heart beats	104557	±14588	97053	(93823–107048)	102712	(96414–116641)	0.079
number of PAC	131	(56–327)	232	(95–1660)	120	(46–253)	0.004
maximum HR	111	±17.8	110	±15.7	112	±18.3	0.694
minimum HR	56	±9.6	52	±6.0	57	±10.2	0.043
average HR	73	±10.9	70	±8.9	74	±11.2	0.143

Data are given as the mean ± SD unless otherwise indicated.

BMI, body mass index; HF, heart failure; HTN, hyper tension; DM, diabetes mellitus; CRF, chronic renal failure; SSS, sick sinus syndrome; SAS, Sleep Apnea Syndrome; ACE-I, angiotensin-converting enzyme inhibitor; ARB, angiotensin II receptor blocker; AAD, antiarrhythmic drugs; LAD, left atrial diameter; LVEF, left ventricular ejection fraction; E/e’, trans-mitral Doppler early ventricular filling velocity to tissue Doppler early diastolic mitral annular velocity; SNRT, Sinus Node Recovery Time; RF, radiofrequency; SVC, superior vena cava; PV, pulmonary vein; ECG, electrocardiogram; PAC, premature atrial contraction; HR, heart rate.

### Sinus node recovery time

**Fig 2:**
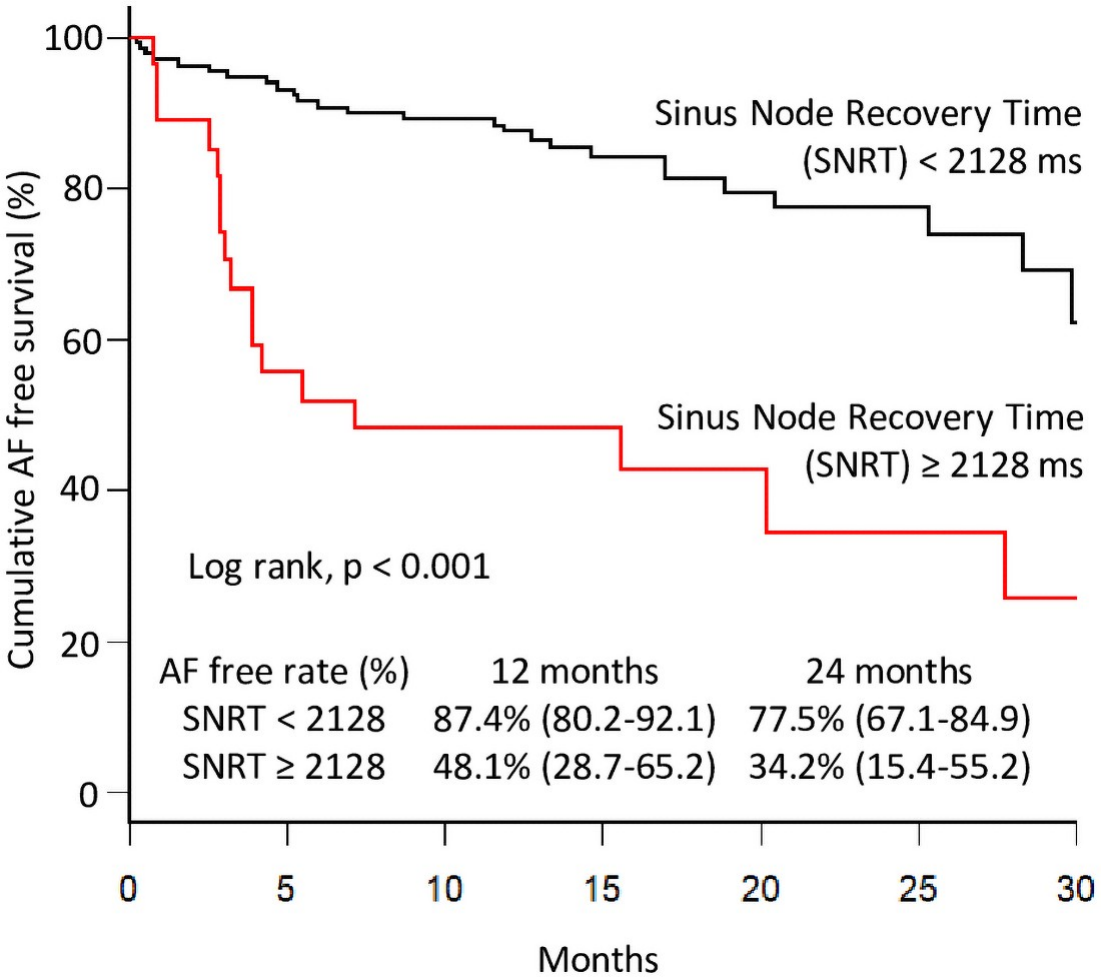
Kaplan–Meier analysis of the atrial fibrillation free survival rate.

Patients with AF recurrence had a longer SNRT (1738 ms vs. 1394 ms, p = 0.012). Using a receiver operating characteristic curve, the area under the curve was 0.6284 (95% confidence interval (CI), 0.520–0.737). The best cut-off value of the SNRT for predicting AF recurrence was 2128 ms (sensitivity 40.0%; specificity 92.0%). Therefore, an SNRT ≥2128 ms was a significant predictor of clinical AF recurrence (hazard ratio (HR) 7.51; 95% CI 2.836–21.264; P<0.001). Using the Kaplan–Meier estimator, the recurrence-free rate was clearly low in patients with an SNRT ≥2128 ms (log-rank, p < 0.001) (Fig 2). Subsequently, the multivariate analysis showed that an SNRT ≥2128 ms was an independent predictor of AF recurrence (HR 7.48; 95% CI 2.94–19.0; P<0.001) (Table 2). There was no significant difference in the use of β-blockers or antiarrhythmic drugs in the multivariate analysis. Additionally, β-blockers or antiarrhythmic drugs had no effect on SNRT (S1 Table).

**Table 2:** Predictors of clinical recurrence.

	Univariate		P value	Multivariate		P value
AADs	2.11	(0.849–5.159)	0.079	1.50	(0.571–3.93)	0.411
β-blockers	0.88	(0.411–1.853)	0.727	0.74	(0.339–1.63)	0.459
Longstanding Persistent	1.73	(0.745–3.961)	0.162	1.78	(0.764–4.16)	0.181
SNRT>2128	7.51	(2.836–21.264)	<0.001	7.48	(2.94–19.00)	<0.001

The abbreviations are the same as those in Table 1.

### Second ablation session

Of the 45 patients with arrhythmia recurrence, 35 (77.8%) underwent a second ablation session. In the second ablation session, we tried as much as possible to induce AF with isoproterenol provocation. However, the trigger for AF was not observed in half the cases. PV, SVC, and non-PV triggers other than SVC were observed in 25.7%, 5.7%, and 31.4% of the patients, respectively. Some patients had more than one trigger. There was no significant difference between PV and SVC triggers for SNRT ≥2128 and SNRT <2128 (14.3% vs. 33.3%, p = 0.262, 0.0% vs. 9.5%, p = 0.506). Although there was no difference in the rate or number of PV reconnections according to the difference in SNRT (78.6% vs. 71.4%, p = 0.712; 2.07% vs. 1.33%, p = 0.090), the ratio of non-PV triggers was clearly higher in the longer SNRT group (57.1% vs. 14.3%, p = 0.012) (Table 3).

**Table 3:** Second ablation session.

	All		SNRT>2128		SNRT<2128		p-value
	N = 35		N = 14		N = 21		
Duration until a recurrence	162	(87, 475)	108	(86, 206)	265	(114, 516)	0.175
Recurrence type							
Atrial fibrillation	24/35	68.6%	8/14	57.10%	16/21	76.20%	0.283
Atrial tachycardia	11/35	31.4%	6/14	42.90%	5/21	23.80%	0.283
PV reconnection	26/35	74.3%	11/14	78.60%	15/21	71.40%	0.712
Number of PV reconnections	1.57	±1.20	2.07	±1.44	1.33	±1.06	0.090
Triggers							
PV trigger	9/35	25.7%	2/14	14.3%	7/21	33.3%	0.262
SVC trigger	2/35	5.7%	0/14	0.0%	2/21	9.5%	0.506
Non-PV trigger	11/35	31.4%	8/14	57.1%	3/21	14.3%	0.012
Unknown	17/35	48.6%	6/14	42.9%	11/21	52.4%	0.733
Procedure							
SVC isolation	17/35	48.6%	5/14	35.7%	12/21	57.1%	0.305
LAPW isolation	29/35	82.9%	12/14	85.7%	17/21	81.0%	1
Non-PV focal ablation	11/35	31.4%	8/14	57.1%	3/21	14.3%	0.012
Defragmentation	7/35	20.0%	3/14	21.4%	4/21	19.1%	1

Data are given as the mean ± SD unless otherwise indicated. PV, pulmonary vein; LAPW, left atria posterior wall.

The abbreviations are the same as in Table 1.

## Discussions

### Main findings

The main findings of this study were as follows: first, the cumulative incidence of AF recurrence was significantly higher in patients with a prolonged SNRT; and second, the prevalence of non-PV triggers was significantly higher in patients with a prolonged SNRT. Similar reports had been published previously [1,7], but our study comprised large number of patients. In addition, we conducted a detailed study of the second ablation after recurrence of AF in this study. To the best of our knowledge, this is the first study to prove the relationship between SNRT and non-PV triggers in patients with persistent AF.

### Relationship between sick sinus syndrome and atrial fibrillation

Sinus node dysfunction is a significant predictor of AF. AF episodes were observed in approximately 40% of patients with sick sinus syndrome, of whom 22% progressed to persistent AF during an 84 month follow-up period [8].

By basic research, sinus node dysfunction itself induces dispersion of the atrial refractoriness, predisposing to atrial ectopy, conduction block, and reentry, thus perpetuating AF [9]. Akoum et al. reported that significant fibrosis in both atria, quantified by LGE-MRI, is associated with clinically significant sick sinus syndrome [3]. These reports indicated that sinus node dysfunction was one of the factors causing AF.

However, Sparks et al. [10] reported significant improvement in corrected SNRT at 3 weeks after the termination of AF. Hocini et al. [11] also noted a significant improvement in sinus node function, with an increase in mean heart rate and a decrease in corrected SNRT, in patients with bradycardia-tachycardia syndrome after catheter ablation of AF. This indicates that sinus node depression is a part of AF-induced electrical remodeling.

Ultimately, AF is a bi-atrial progressive fibrotic disease, closely associated with sinus node dysfunction. We evaluated the effect of SNRT in asymptomatic patients with sinus node dysfunction rather than classical SNRT; thus, the clinical meaning differed from that of previous studies. Nevertheless, we demonstrated that prolonged SNRT is a clinically useful parameter for predicting underlying sinus node dysfunction and AF recurrence after catheter ablation in patients with persistent AF.

### Importance of triggers in persistent atrial fibrillation

The substrate is regarded as a key material factor in persistent AF, and various mechanisms of reentry have been proposed: circus movement reentry, the leading circle concept, spiral wave reentry, and the multiple wavelet hypothesis [12]. The reason PV isolation has beneficial effects on persistent AF is considered to be associated with the location of the drivers. One study found the AF drivers were located near the PVs or left atrial roof in 50% of patients with paroxysmal AF and 33.5% of patients with persistent AF. Of note, this study also reported that 45% of the rotors or AF drivers were coincidentally ablated during the empiric lesion set delivered with the goal of PV isolation [13]. Thus, it is conceivable that PV isolation is effective not because of the isolation of PV triggers but because of the elimination of AF maintenance drivers.

However, the importance of the trigger has also been reported even in persistent AF. Inoue et al. [14] reported that a trigger-based mechanism plays one of the major roles in AF persistence. They described an immediate recurrence of AF after electric cardioversion as an IRAF (Immediate Recurrence of AF) and found that the clinical outcome of AF ablation was strongly influenced by the successful elimination of IRAF triggers. The importance of non-PV triggers has also been reported after catheter ablation [15,16]. Kurotobi et al. [17] reported that the incidence of non-PV foci, number of non-PV foci, incidence of foci in the right atria, and incidence of multiple foci were significantly higher in persistent than in paroxysmal AF. Takigawa et al. [18] reported that non-PV foci may be associated with atrial remodeling, and the presence of non-PV foci results in a worse outcome after catheter ablation. Kato et al. [19] reported that catheter ablation of non-PV foci is effective when they are mappable; however, multi-changing non-PV foci are associated with a worse prognosis. These reports indicate that unsuccessful mapping and ablation of non-PV triggers are associated with an extremely high recurrence rate of AF and highlight the clinical importance of non-PV triggers, especially in persistent AF, together with the role residual non-PV triggers play in AF recurrence. In our study, one-third of the patients with recurrence had non-PV triggers, and 58% of the patients with prolonged SNRT had non-PV triggers. Non-PV triggers were significantly higher in this group, reflecting the extent of atrial remodeling, which may cause non-PV triggers.

### Effectiveness of SNRT in catheter ablation

Paroxysmal AF patients with sick sinus syndrome are at a higher risk of AF recurrence after catheter ablation, and non-PV triggers are associated with AF recurrence [20]. To the best of our knowledge, this is the first study to prove the relationship between SNRT and non-PV triggers in patients with persistent AF. Normally, it is difficult to evaluate sinus node dysfunction in patients with persistent AF. We assessed the degree of sinus node dysfunction by measuring SNRT when AF was terminated during catheter ablation. Patients who had a prolonged SNRT had a high recurrence rate and a high ratio of non-PV triggers in the second ablation.

Sinus node disease is associated with diffuse atrial remodeling characterized by structural changes, conduction abnormalities, and increased right atrial refractoriness [5]. Akoum et al. [3] reported that significant fibrosis in the right atria, quantified using LGE-MRI, is associated with clinically significant sick sinus syndrome. Furthermore, the right atrial fibrosis and left atrial fibrosis were also correlated. Chang et al. [4] reported a relationship between sinus node dysfunction and voltage map. They reported that regional atrial remodeling near the sinus node area in the right atria is associated with sinus node dysfunction. In Chang’s study, patients with sinus node dysfunction tended to have a lower left atrial mean peak-to-peak voltage.

These results indicate that atrial electrical remodeling progresses globally and that SNRT represents the extent of entire atrial remodeling. We found no relationship between PV reconnection rates and prolongation of the SNRT, but the ratio of recurrences and non-PV triggers was higher in the prolonged SNRT patients. We hypothesize that in patients with prolonged SNRT, diffuse remodeling of the atria progresses, which induces AF recurrence via non-PV triggers. Therefore, in addition to PV isolation, erasure of non-PV triggers is required to eliminate AF in these patients.

Persistent AF involves many mechanisms. Eliminating persistent AF requires personalized therapy for each mechanism rather than a standardized method. Prolongation of the SNRT might indicate the existence of non-PV triggers as inducers of AF.

Hence, measurement of SNRT might be used for the stratification of treatments for patients with persistent AF.

### Study limitations

This study had several limitations. First, it was a single-center, retrospective, observational study. Second, inducing non-PV AF triggers under an isoproterenol infusion and burst pacing/cardioversion protocol may induce non-clinical AF triggers or unmask clinical AF triggers. Third, despite efforts to perform frequent 24-hour Holter monitoring or event recording in any patient suspected of having an AF recurrence, completely asymptomatic patients with nocturnal or short AF episodes could have been missed. Fourth, β-blockers and antiarrhythmic drugs were continued in some patients, and pharmacological autonomic blockers such as atropine and propranolol were not used, which may affect SNRT.

## Conclusions

Patients with prolonged SNRT had a high prevalence of AF recurrence and non-PV triggers, indicating the existence of non-PV triggers as inducers of AF. Measuring SNRT might be used for the stratification of patients with persistent AF.

## Supporting information

S1 Table Relationship between SNRT and medication. (DOCX)
