# Supplementary material for: Impact of the sinus node recovery time after termination of atrial fibrillation during catheter ablation on clinical outcomes in patients with persistent atrial fibrillation
Source: PLoS One. 2021 Nov 5;16(11):e0259750. doi: 10.1371/journal.pone.0259750 (PMC8570470; doi:10.1371/journal.pone.0259750)
Supplement: S1 Table — (DOCX) [file pone.0259750.s001.docx]

Supplemental Table1. Relationship between SNRT and medication

ACE-I, angiotensin-converting enzyme inhibitor; ARB, angiotensin II receptor blocker; AADs, antiarrhythmic drugs; SNRT, Sinus Node Recovery Time

|  |  | SNRT (msec) | p-value |
| --- | --- | --- | --- |
| ACE-I, ARB | (+) | 1541(1305-1900) | 0.112 |
|  | (-) | 1393(1218-1886) |  |
| βblockers | (+) | 1447(1231-1886) | 0.811 |
|  | (-) | 1463(1215-1948) |  |
| AADs | (+) | 1447(1291-2484) | 0.349 |
|  | (-) | 1447(1225-1855) |  |
